# Virulence Evolution via Pleiotropy in Vector‐Borne Plant Pathogens

**DOI:** 10.1002/ece3.70741

**Published:** 2024-12-15

**Authors:** Elise Woodruff, Nate B. Hardy

**Affiliations:** ^1^ Department of Entomology and Plant Pathology Auburn University Auburn Alabama USA

## Abstract

The dynamics of virulence evolution in vector‐borne plant pathogens can be complex. Here, we use individual‐based, quantitative‐genetic simulations to investigate how virulence evolution depends on genetic trade‐offs and population structure. Although quite generic, the model is inspired by the ecology of the plant‐pathogenic bacterium *Xylella fastidiosa* , and we use it to gain insights into possible modes of virulence evolution in that group. In particular, we aim to sharpen our intuition about how virulence may evolve over short time scales via antagonistically pleiotropic alleles affecting pathogen performance within hosts and vectors. We find that even when pathogens find themselves much more often in hosts than vectors, selection in the vector environment can cause correlational and potentially non‐adaptive changes in virulence in the host. The extent of such correlational virulence evolution depends on many system parameters, including the pathogen transmission rate, the proportion of the pathogen population occurring in hosts, the strengths of selection in host and vector environments, and the functional relationship between pathogen load and virulence. But there is a statistical interaction between the strength of selection in vectors and the proportion of the pathogen population in hosts, such that if within‐vector selection is strong enough, over the short term, it can dominate virulence evolution, even when the host environment predominates.

## Graphical abstract

In general, natural selection is more efficient in more commonly encountered habitats. Here, with evolutionary simulations, we show that even when vector‐borne pathogens much more often occur within hosts, correlational and potentially maladaptive virulence evolution can be driven by selection in vectors.

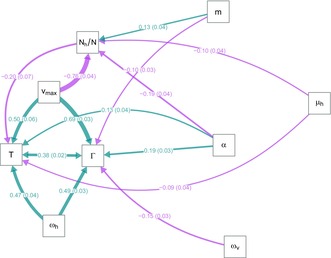

## 1 Introduction

In a mixed environment, selection tends to be more efficient in habitat types that are more commonly encountered or productive (Via and Lande 1985; Whitlock 1996; Draghi 2021). Therefore, as long as a population is adapting to a common or productive habitat, evolution in less common or productive habitats is expected to be largely correlational (Via and Lande 1985; Hardy and Forister 2023). But what if a population's life history entails obligate movements through habitats of different prevalence or productivity? And what if a population's evolution can alter the prevalence and quality of habitat types, for example, via over‐exploitation (Crossan, Paterson, and Fenton 2007)? Here we consider these general questions, with a special focus on virulence evolution in vector‐borne plant pathogens.

For vector‐borne pathogens, the within‐host environment is much more commonly encountered and productive than the within‐vector environment; hosts live longer, and can carry a higher pathogen load. So, without accounting for the details of their life history, we might expect the evolution of virulence‐affecting pathogen phenotypes to be driven by selection in the host, and any evolutionary change in the vector to be largely correlational (Via and Lande 1985; Gandon 2004; Osnas and Dobson 2012; Hardy and Forister 2023). But vector‐borne transmission could change that expectation, as it entails obligate alternations between host and vector environments, and pathogen‐induced host mortality can reduce disparities in the prevalence and quality of host and vector environments (Crossan, Paterson, and Fenton 2007; Evensen, White, and Boots 2024).

To improve our intuition about how vector‐borne dispersal affects asymmetries in selection across habitat types, we develop and analyze individual‐based simulation models. Our models are based on the trade‐off theory of virulence evolution, which predicts an optimal level of virulence that balances short‐ and long‐term transmission efficiency (Anderson and May 1982; Ewald 1983; Frank 1996; Alizon et al. 2009; Bull and Lauring 2014; Cressler et al. 2016). In general, short‐term transmission probability increases with pathogen load (Bull, Molineux, and Rice 1991; Ni and Kemp 1992; Ebert and Mangin 1997; Day 2001). But high pathogen load can increase host mortality. This limits the time over which an infected host can be the source for pathogen transmission to a new host, and causes a negative meta‐population‐level negative feedback on virulence evolution (Dobson 1990; Alizon et al. 2009; Bull and Lauring 2014). This theory has been developed and explored primarily with adaptive‐dynamics analysis (Geritz et al. 1998; Dieckmann 2005) of epidemiological compartment models that are not explicitly population genetic (Regoes, Nowak, and Bonhoeffer 2000; Day and Proulx 2004; Best, White, and Boots 2009; Osnas and Dobson 2012; Williams 2012; Auld, Searle, and Duffy 2017). Such studies can tell us about equilibrium conditions, but not about how long a system might take to arrive at an equilibrium, or what might happen along the way (Day and Proulx 2004). Here, our aim is to understand how the non‐equilibrium dynamics of virulence evolution depend on genetic architecture and population structure. An individual‐based simulation approach is well‐suited for that purpose.

Our model is inspired by several cases in which the emergence of virulent plant pathogen genotypes has been associated with the arrival of a new vector species or genotype. For example, the global spread of the *Bemisia tabaci* is thought to have repeatedly driven genetic divergence and virulence evolution in begomoviruses, many of which now cause serious diseases problems in crops ranging from okra in western Africa to tomato in Peru and Taiwan (Gilbertson et al. 2015). Likewise, the re‐emergence of Pierce's disease in California vineyards in the 1990's was co‐incident with the spread of a new vector species, the glassy‐winged sharpshooter (Hemiptera: Cicadellidae: *Homalodisca vitripennis*) (Hopkins and Purcell 2002). In this case, three observations lead us to suspect that selection within vectors might have driven correlational evolution of virulence within hosts: (1) *H. vitripennis* is an exceptionally inefficient vector; a glassy‐winged sharpshooter has a low probability of acquiring *X. fastidiosa* from an infected host and transmitting it to a susceptible host (Redak et al. 2004). (2) Nevertheless, as it has become numerically dominant, *H. vitripennis* has come to account for most transmission to and from grapevine in California. (3) Much of the harm *X. fastidiosa* causes to its hosts has been attributed to the plastic expression of “sticky” cell phenotypes which can clog xylem vessels, but also increase the efficiency of vector acquisition (Chatterjee, Wistrom and Lindow 2008; Killiny and Almeida 2014). Can selection for improved within‐vector performance cause correlational and potentially non‐adaptive evolution of virulence in hosts?

## 2 Methods

To address this question, we simulate the evolution of a structured meta‐population of pathogen individuals in an environment composed of a mix of host and vector habitat patches, with pathogen migration permitted only between patches of a different type, that is, from a host patch to a vector patch, or vice versa. This constraint on pathogen migration distinguishes our model from previous models of virulence evolution in heterogeneous host environments (Regoes, Nowak, and Bonhoeffer 2000; Day 2003; Gandon 2004; Osnas and Dobson 2012; Evensen, White, and Boots 2024). Another point of departure is how we model life history trade‐offs between habitat types. Whereas previous studies have tended to assume that such trade‐offs are fixed (Regoes, Nowak, and Bonhoeffer 2000; Day 2003; Osnas and Dobson 2012), we express them as statistical tendencies in the distribution of quantitative allele effects. More specifically, we assume that two quantitative pathogen phenotypes—one determining pathogen performance within hosts, and the other determining performance within vectors—are subject to mutations that tend to be antagonistically pleiotropic (Via and Lande 1985; Hardy and Forister 2023). Note that although loosely inspired by *X. fastidiosa* , we do not attempt to strictly parameterize the model in accordance with any empirical system. Instead, we explore simplified systems that capture key pathosystem features, and we conduct a sensitivity analysis to characterize the robustness our inferences to variation in those features.

In our model, each individual has a haploid, single‐chromosome, 50 kb genome, and reproduces clonally, without recombination. At the start of each simulation, the population is genetically uniform; genomes are essentially empty containers for future mutations. The pathogen population is divided into *n*_*d*_ = 100 demes, each of which occurs in either a host or vector patch. The model parameter *ρ* = 0.1 gives the proportion of patches that are hosts; in comparison to vector patches, host patches are less abundant, but more productive.

In each pathogen generation, pathogen demes suffer extirpation at background mortality rates *μ*_*v*_ {0.05} per vector patch and *μ*_*h*_ {0.0001–0.01} per host patch. This reflects that in nature hosts (e.g., a grapevine) tend to live longer than vectors (e.g., a sharpshooter). In hosts, this rate can be elevated by a virulence effect. In general, pathogen load and the virulence of an infection are positively correlated (Bull, Molineux, and Rice 1991; Ni and Kemp 1992; Ebert and Mangin 1997; Day 2001; Longdon et al. 2015). We express this relationship with a logistic function, *v*_*i*_ = *v*_*max*_/(1 + exp.(−α*d_*i*_)), where *v*_*i*_ is the excess risk of mortality experience by host *i*, *v*_*max*_ is the maximum possible virulence effect {0.01–0.99}, *d*_*i*_ is the density of pathogens within host *i*, that is *N*_*i*_/*K*_*i*_, and *α* {0.1–10.0} controls the steepness of the logistic mapping of pathogen density to virulence. Using a logistic function ensures that the virulence phenotype is correctly bounded between zero and 1‐*μ*_*h*_. As *α* approaches zero, the function becomes increasingly linear. Patch replacement is instantaneous. Vector or host death amounts to setting the number of pathogens in that particular patch to zero. The patch is then immediately available for re‐colonization in the next pathogen generation.

The pathogen life cycle begins with clonal offspring production, with each individual producing one offspring individual. Generations are overlapping. Offspring genomes are generated by random mutation of the parental genome. The mutation rate is 1e‐7 per site, per genome, per generation. Mutations affect two quantitative phenotypes: a within‐host performance phenotype and a within‐vector performance phenotype. (These phenotypes affect fitness later in the life cycle, after possible migration between habitat patches. Details are given below.) When a mutation occurs, a two‐dimensional vector of allele effects is drawn from a zero‐meaned random bivariate normal distribution with variances of 1.0, and symmetrical covariances, *κ* {−0.2, −0.8}, the sign and magnitude of which controls the pleiotropy between the two phenotypes (as in Hardy and Forister 2023). An individual's within‐host performance phenotype is determined by the sum of the first elements of allele effect vectors. Likewise, an individual's within‐vector performance phenotype is the sum of the second elements of allele effect vectors. With means of zero for the bivariate normal distribution, there are no directional biases of allele effects on phenotypes. The variance terms have meaning only in relation to the distances between a population's starting values for the within‐host and within‐vector performance phenotypes [0, 0] and the optima for those phenotypes [5.0, 5.0]; by setting effect variances to be less than those distances, we assert that adaptive paths will entail a sequence of several mutational steps. If *κ* > 0, positive pleiotropy prevails and an allele that increase the host‐performance phenotype value tends to also increase the vector‐performance phenotype value. Conversely, when *κ* < 0, antagonistic pleiotropy prevails. Here we are especially curious about the case of strong antagonistic pleiotropy between phenotypes affecting pathogen performance in vectors and hosts, and therefore we focus on *κ* = −0.8.

**FIGURE 1:**
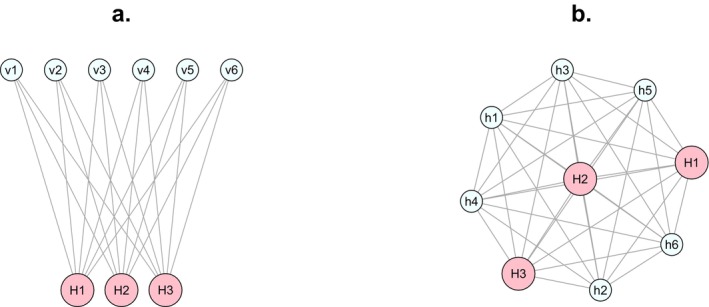
Alternative population structures. Network vertices represent specific host (*H*_*i*_ or *h*_*i*_) or vector (*v*_*i*_) patches. Larger vertices have higher pathogen carrying capacities. Edges represent possible paths for pathogen migration. (a) In the main model, the pathogen population is vector‐borne; migration is only possible between trophic levels, that is, from a host (*H*_*i*_) to a vector (*v*_*i*_), or vice versa. (b) In an alternative version of the model, migration is unfettered; thus, rather than consisting of a mix of hosts and vectors, the environment consists of a few large and susceptible hosts (*H*_*i*_) and several small and tolerant hosts (*h*_*i*_).

The next step in the life cycle is migration, that is, pathogen transmission. This happens at per capita rate *m* {0.001–0.2} and, in the main version of the model (Figure 1a) is random between patches except that migrants from a host patch can only move to a vector patch and vice versa. To isolate the effects of transmission mode, in an alternative version of the model (Figure 1b), we relax this constraint and let migration be unfettered between patches. In every other respect, the models are the same. Without vector transmission, the model describes the evolution of a population in an environment in which there are two kinds of hosts, one being large, rare and susceptible to infection, and the other being small, abundant and tolerant of infection. Comparison of the pathogen evolutionary dynamics in this unfettered‐migration model to the main vector‐born model, reveals the effects of vector transmission per se.

After migration comes selection and population regulation. This entails genotype‐environment matching, and negative density dependence. Selection is hard; it affects survival and has demographic effects. In the vector environment, the match between a pathogen's within‐vector‐performance phenotype and the within‐vector optimum determines their viability, that is, their probability of surviving until reproduction. This matching is via a standard Gaussian fitness function (Bürger and Lynch 1995; Scheiner and Holt 2012; Scheiner 2013), with variance *ɷ*_*v*_ {1.0–10.0} setting the weakness of selection. Likewise, in the host environment, viability is determined by the match between a pathogen's within‐host performance phenotype and the within‐host optimum, using a similar Gaussian fitness function, but with variance *ɷ*_*h*_ {1.0–10.0}. In vector and host patches, individual‐level fitness is also density dependent; in habitat patch *i*, each individual's viability is rescaled by the ratio of the patch carrying capacity *K*_*i*_ and the current local pathogen population size, *N*_*i*_. As described above, in hosts, there is also group‐level selection via a virulence effect. As populations evolve mean within‐host performance phenotype values that more closely match the optimum and mean within‐host fitness increases, so too does the probability of host death and deme extirpation.

After selection, the life cycle starts again with offspring production.

At the start of each simulation, pathogens are monomorphic, with a value of zero for both of their phenotypes, and the optimal value for each of these phenotypes is set to five phenotypic units. During the first 200 generations, the pathogen population is subject only to density‐dependent regulation; selection and virulence effects are not applied, and so genetic diversity accumulates. At generation 201, selection and virulence commence. We then follow the population as it adapts to its host and vector environments. Our view of these dynamics is based on two test statistics. The first, *T*, is simply a long‐transformed count of how many generations it takes to evolve a mean host‐performance phenotype within 10% of the optimum, and thus cause near‐maximum virulence. Note that because of the negative meta‐population‐level feed‐backs induced by high virulence, such proximity to the optimum host value might not be the equilibrium state of a pathosystem. Therefore, *T* is best interpreted as the hazard of evolving high virulence, even if only temporarily.

**FIGURE 2:**
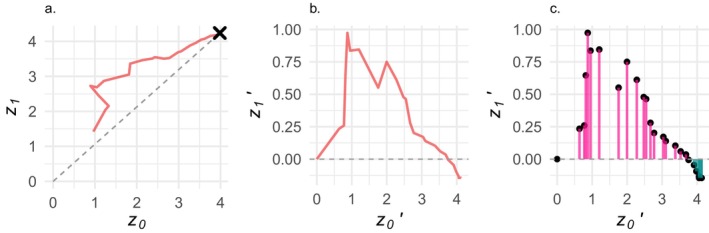
Calculation of the *Γ* statistic. (a) An example evolutionary path through the phenotype space. For this simulation *κ* = −0.2, *v*_*max*_ = 0.3, *m* = 0.1, *K*_*v*_ = 20, *K*_*h*_ = 2000, *ɷ*_*v*_ = 3.0, *ɷ*_*h*_ = 3.0, *μ*_*h*_ = 1e‐4, *μ*_*v*_ = 0.1, *α* = 5.0, and *ρ* = 0.1. (b) That same path translated to start at the origin and rotated so that the ideal path from the origin to the joint phenotypic optimum lies along the x‐axis. (c) *Γ* is calculated as the sum of deviations from the ideal path, scaled by the length of the path in generations. When *Γ* is positive, the evolutionary path bends mostly toward the vector environment; conversely when it is negative, the host environment dominates.

The second statistic we track, *Γ*, is a measure of how, until a population evolves a within‐host performance phenotype within the 10% threshold of the optimum, the population's evolutionary path deviates from the theoretical ideal path; it expresses the extent to which adaptation in the pathogen population is dominated by the vector or host habitat type (See Figure 2 for an example).

To simplify the calculation of *Γ*, we first translate a population's evolutionary path through the phenotypic space so as to start at the origin. We do this by subtracting the first post‐burnin (generation 201) mean value for each phenotype (*z*_0_, and *z*_1_) from the mean phenotype value for each subsequent generation. Since the optimal value for each phenotype is five, and pathogen populations start out with phenotype values of zero, a straight evolutionary path to the joint optimal phenotype would have a slope of one. Therefore, for each simulation, we rotate the translated evolutionary path *D* radian degrees about the origin, where *D* is the inverse tangent of one. This rotation is done as follows:

$${z_{0i}}^{\prime }=z_{0i}*\cos\left(D\right)+z_{1i}*\sin\left(D\right);{z_{1i}}^{\prime }=z_{1i}*\cos\left(D\right)-z_{\text{0i}}*\sin\left(D\right)$$

where (*z*_0*i*_, *z*_1*i*_) is point *i* along the simulated post‐burnin translated evolutionary path, *z*_0*i*_ is the population's mean value for the host performance phenotype, *z*_1*i*_ is the mean value for the vector performance phenotype, and (*z*_0*i*_′, *z*_1*i*_′) is that same point in the rotated coordinate space. We can then calculate the degree to which the evolutionary path bends toward what is optimal in the vector environment as sum (*z*_1*i*_′)/*T*, the per‐generation average deviation from the ideal evolutionary path.

A total of 200 simulations were performed for each version of the model, that is, the vector‐borne transmission model, and the unfettered‐migration model, and for each value of *κ* {−0.2, −0.8}. For each run, a value for each other free model parameter was drawn from a random uniform distribution with ranges as given in Table 1.

**TABLE 1:** Model parameters and variables.

Parameter	Definition	Range of values
*n*_*d*_	Number of pathogen demes	100
*ρ*	Relative frequency of host environments	0.1
*κ*	Strength of pleiotropic covariance	*κ =* −0.2, −0.8
*ω*_*h*_	Weakness of selection in host	1 < *ω*_*h*_ < 10
*ω*_*v*_	Weakness of selection in vector	1 < *ω*_*v*_ < 10
*m*	Migration rate	0.001 < *m* < 0.2
*μ*_*h*_	Background rate of host mortality	1e‐4 < *μ*_*h*_ < 0.01
*μ*_*v*_	Rate of vector mortality	0.05
*v*_*max*_	Maximum virulence effect	0.1 < *v*_*max*_ < 0.99
*α*	Steepness of the mapping of pathogen load to virulence	0.1 < *α* < 10.0
*K*_*h*_	Pathogen carrying capacity of one host	200 < *K*_*h*_ < 2000
*K*_*v*_	Pathogen carrying capacity of one vector	20

Variable	Definition	Range of value
*T*	Log transformed number ‐of generations until mean pathogen within‐host‐performance phenotype within 10% of optimum	201 < *T* < 1e4
*Γ*	Degree to which pathogen population's evolutionary path bends toward to the optimum in the vector environment	‐inf < *Γ* < inf
*N*_*h*_	Number of pathogen individuals in host patches	0 ≤ *N*_*h*_
*N*_*v*_	Number of pathogen individuals in vector patches	0 ≤ *N*_*v*_

We analyzed simulation model outputs by fitting multivariate linear models, using the R package *lme4* (Bates 2003). In one model, the response variable was *T*. In the other, the response was *Γ*. For both models the fixed predictor variables were (1) *ɷ*_*h*_, the weakness of selection in hosts, (2) *ɷ*_*v*_, the weakness of selection in vectors, (2) *m*, the migration rate, (3) *μ*_*h*_, the background rate of host mortality, (4) *v*_*max*_, the maximum extent of virulence, that is, pathogen‐induced host mortality, (5) *α*, the steepness of the mapping of pathogen load to virulence, (6) *N*_*h*_/*N*, the log‐transformed proportion of pathogens in hosts, and (7) the interaction between *N*_*h*_/*N* and *ɷ*_*v*_. This interaction term gives us an especially pertinent measure of the possibility that selection for improved within‐vector performance could drive the correlational evolution of virulence in hosts, even if most of the pathogen populations occurs within hosts. It reveals how the effect of selection within vectors on adaptation within hosts depends on the predominance of the host environment.

To get a better sense for what could be complex causal links in the system, we also analyzed model outputs by fitting a structural equation model, using the R package *laavan* (Rosseel 2012).

To sum up, we examined how, when vectors are more abundant but less productive than hosts, and there is antagonistic pleiotropy between within‐host and within‐vector performance phenotypes (1) the time it takes a population to evolve a host‐performance phenotype close to the optimal value, and (2) the degree to which a population's evolutionary path through the phenotypic space bends toward or away from the vector environment depends on (a) the relative strengths of selection in host and vector demes, (b) the migration rate, (c) the shape of the mapping of pathogen load to virulence, and (d) the maximum virulence effect of high density in host demes. We also considered how all of this is affected by doing‐away with vector‐based transmission, and allowing for completely random migration.

Simulations were performed with the Selection of Linked Mutations (SLiM) v.4 framework (Haller and Messer 2023). SLiM facilitates the development of forward‐time individual‐based evolutionary simulation models by providing users with a hierarchical set of general model components that mirrors the natural hierarchy of biological organization (e.g., genomes, individuals, and demes), along with general routines for population genetic processes (e.g., mutation, recombination, and selection). SLiM models are specified with codes written in the Eidos language. The Eidos code for the model described here is provided in Data S1.

## 3 Results and Discussion

**FIGURE 3:**
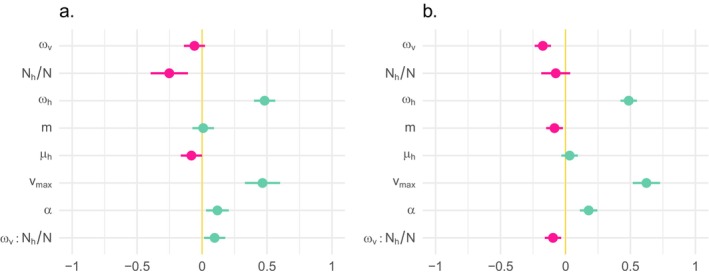
Summary of multivariate linear models decomposing the variance in (a) *T*, the log‐transformed number of generations it takes for the pathogen population to evolve a within‐host performance phenotype within 10% of the optimum, and (b) *Γ*, the degree to which, before *T*, the pathogen population's evolutionary path bends toward (*Γ* > 0) or away from (*Γ* < 0) the vector environment. Points show positive (teal) and negative (magenta) estimated coefficients, and horizontal bars show 95% confidence intervals. All predictors have been centered and scaled to units of standard deviations (the units of the x‐axis).

**FIGURE 4:**
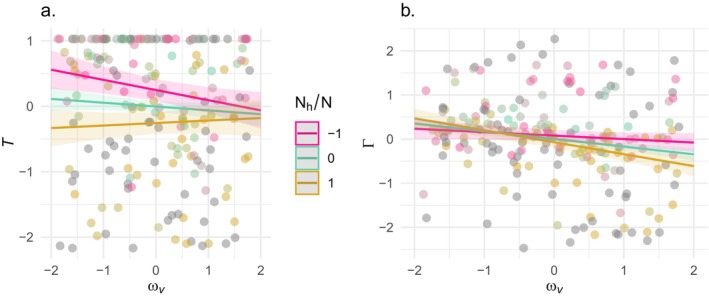
Marginal effect estimates of the interaction between the weakness of selection in vectors, *ω*_*v*_, and the predominance of hosts *N*_*h*_/*N*. Each point and linear interpolation shows the predicted value for *T* (a) and *Γ* (b) for a given value of *ω*_*v*_ in combination with a level of *N*_*h*_/*N*, where magenta is for −1 SD, teal is for the mean, and gold is for +1 SD.

Let us first consider variation in *T*, which measures the time it takes for the pathogen population to evolve a host‐performance phenotype close to the optimum, and thus high virulence. Prior to analysis, the data were mean‐centered and variance‐scaled, so effects are expressed in units of standard deviations (SD). When there is strong antagonistic pleiotropy (*κ* = −0.8; adjusted*‐R* ^*2*^ = 0.70; Figure 3a.), two primary fixed effects significantly decrease *T*. The first is *μ*_*h*_, the background rate of host mortality (coefficient = −0.08 SD; *p* = 0.049); this aligns with previous theoretical work showing that, in homogeneous host environments, increasing the background rate of host mortality causes an increase in the equilibrium level of virulence (Kakehashi and Yoshinaga 1992; Lenski and May 1994; Day 2001), although this need not be so in heterogeneous host environments (Williams and Day 2008; Williams 2012). The second is *N*_*h*_/*N*, the preponderance of the host environment (coefficient = −0.25 SD, *p* = 6.6e‐4); when the host environment is more predominant, pathogens more rapidly evolve within‐host performance phenotypes that closely match the within‐host optimum (Whitlock 1996; Osnas and Dobson 2012). Four parameters significantly increase *T*, that is, delay the evolution of near‐optimal within‐host performance phenotypes. The first is *v*_*max*_, the maximum additional host mortality than can be caused by an infection (coefficient = 0.46 SD, *p* = 1.6e‐10). The second is *α*, the steepness of the mapping of pathogen load to virulence (coefficient = 0.12; *p* = 8.1e‐3). Increasing either of these parameters increases the meta‐population‐level fitness cost of evolving optimal within‐host performance phenotypes, and is therefore expected to push the equilibrium within‐host performance phenotype away from the optimum. The third parameter that increases *T* is *ɷ*_*h*_, the weakness of selection in host patches (coefficient = 0.48 SD; *p* < 2‐e16); evolving optimal within‐host performance phenotypes is slower when the within‐host fitness consequences of maladaptation are less pronounced. The fourth is *ɷ*_*v*_:*N*_*h*_/*N*, the interaction between the weakness of selection in vectors, and the predominance of pathogens in hosts (coefficient = 0.097, *p* = 0.02) (Figure 4a.). Since the overall mean effect of the weakness of selection in vectors on *T* is negative, this positive interaction means that, as the host environment becomes more predominant, the importance of selection in the vector is diminished. Therefore, in natural communities where pathogen individuals occur at much higher frequency in hosts, antagonistic pleiotropy between within‐host and within‐vector performance is unlikely to have a strong effect on the time it takes to evolve high virulence.

With less antagonistic pleiotropy, we have less of a basis for expecting selection in vectors to drive non‐adaptive correlational evolution of the within‐host performance phenotype. With a more even distribution of pleiotropic allele effects, there is a higher probability of general‐vigor mutations that can move both phenotypes toward their optima. Indeed, when we set the antagonistically pleiotropic covariance, *κ*, to −0.2, it accelerates pathogen adaptation to the host environment. Across simulations, the mean of the antilog of *T* is 3641 generations when *κ* = −0.8, and 2208 generations when *κ* = −0.2, a 40% reduction. Beyond that, reducing antagonistic pleiotropy has two main effects on evolutionary model dynamics: (1) the negative effects of virulence on *T* become more pronounced (*v*_*max*_ coefficient = 0.83; *α* coefficient = 0.21), and (2) the effects of all other statistical model covariates are diminished. Strong antagonistic pleiotropy can impede joint optimization of the two performance phenotypes. Without such genetic constraints, the ecological constraints on phenotype evolution (*v*_*max*_) become more important.

Because of the negative meta‐population‐level feed‐back, a close match between the mean host‐performance phenotype and the optimum can be nonadaptive, and therefore, reaching the host‐habitat‐match threshold could be largely dictated by stochastic processes. This complicates the interpretation of variation in *T* and makes our alternative statistic *Γ* especially useful.

So, let us turn to the analysis of the variance in *Γ*, a measure of the degree to which simulated evolutionary paths bend toward (*Γ* > 0) or away from (*Γ* < 0) the vector environment. With *Γ*, statistical models are consistent across variation in the strength of negative pleiotropy. For brevity, we discuss only the results for *κ* = −0.8 (adjusted *R* ^2^ = 0.82). As shown in Figure 3b, *ω*_*h*_ (coefficient = 0.49; *p* < 2e‐16), *v*_*max*_ (coefficient = 0.63; *p* < 2e‐17), and *α* (coefficient = 0.18; *p* < 5.21e‐7) significantly increase *Γ*. This indicates that weaker selection in hosts and higher levels of virulence push the pathogen's evolutionary path toward optimization of within‐vector performance. However, three first‐order‐fixed effects significantly decrease *Γ*: (1) *ɷ*_*v*_ (coefficient = −0.17, *p* < 1.38e‐7); (2) *m* (coefficient = −0.08; *p* = 0.01), and *N*_*h*_ */N* (coefficient = −0.1; *p* = 3.0e‐3). This indicates that weaker selection in the vector, higher per‐capita transmission rates, and a more predominant host environment cause the evolutionary path to veer toward optimization of within‐host performance. Now, for a critical point: in comparison to what we saw for *T*, with *Γ* the interaction between the weakness of selection in the vector and the predominance of the host, *ɷ*_*v*_:*N*_*h*_/*N*_*v*_ , reverses sign (coefficient = −0.096; *p* = 2.3e‐3). Since the overall mean effect of the weakness of selection on *Γ* is negative, a negative interaction with *N*_*h*_/*N* means that as the host environment becomes more predominant, selection in vectors actually becomes more important. We can explain this as arising from a mix of neutral and non‐neutral processes. When vectors are more abundant, there is a higher probability that a few pathogens that are poorly adapted to the vector environment will nonetheless survive the trip from one host to another. Conversely, when vectors are rare, they constitute a more constrictive transmission bottleneck, and sustained transmission depends more strongly on adaptation to the vector environment.

To summarize, when the host habitat is more commonly encountered, the rate of pathogen adaptation to the host environment (*T* ^−1^) increases, and the importance of selection in the vector environment decreases (Figure 4a.). But as selection in the vector strengthens, a pathogen population's evolutionary path to the joint performance phenotype optimum deviates more toward the optimum in the vector environment, and this bias increases with the predominance of the host environment (Figure 4b).

How much do these inferences depend on vector‐dispersal as opposed to heterogeneity across pathogen environments? To isolate the effects of dispersal mode, we ran two batches of simulations, one with migration constrained to be between host and vector habitat types, and another with unfettered migration. In both batches, we assumed strong antagonistic pleiotropy (*κ* = −0.8) and a moderately steep mapping of pathogen load to virulence (*α* = 5.0). We combined the outputs of both model types, and fit linear models which included terms for the interaction between dispersal mode, *δ*, and each other predictor.

**FIGURE 5:**
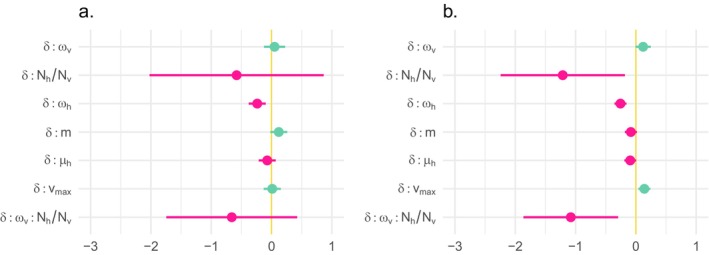
Interactions between dispersal mode (*δ*) and other predictors of (a) *T*, the number of generations until a pathogen population evolves a within‐host performance phenotype within 10% of the optimum, and (b) *Γ*, before *T*, the degree with which correlation evolution in the pathogen population is dominated by the vector (*Γ* > 0) or host (*Γ* < 0) environment. Points show the positive (teal) and negative (magenta) coefficients, and horizontal lines give 95% confidence intervals.

For *T* (Figure 5a), the interactions between *δ* and each other model parameters are insignificant, except for that between *δ* and *ω*_*h*_ (coefficient = −0.24 SD, *p* = 0.001); the only significant effect of switching to unfettered migration is an increase in the importance of selection in susceptible host environment. In contrast, for *Γ*, *δ* has significant interactions with four other model covariates (Figure 5b): (1) With vector‐borne dispersal, weakening selection in hosts has a positive effect on *Γ* (Figure 3b). A negative interaction between dispersal mode and *ω*_*h*_ (coefficient = −0.26 SD, *p* = 6.2e‐7) indicates that this effect is diminished by unfettered migration. Although it is just shy of significance (coefficient = 0.12; *p* = 0.062), we find a similar diminishing interaction between dispersal mode and the weakness of selection in vectors, *ω*_*v*_. (2) With vector‐borne dispersal, *v*_*max*_ has a strong positive effect on *Γ*. A positive interaction between *v*_*max*_ and *δ* (coefficient = 0.14 SD, *p* = 5.6e‐3) indicates that its effect is amplified with unfettered migration. (iii) With vector‐born dispersal, *N*_*h*_/*N* has a strong negative effect on *Γ*. As for *v*_*max*_, this effect is amplified with unfettered migration (*δ*:*N*_*h*_/*N* coefficient = −1.21 SD, *p* = 0.021). (iv) With vector‐borne dispersal, increasing the preponderance of the host environment increases the impact of selection in vectors on *Γ*. A negative interaction between delta and *ω*_*v*_:*N*_*h*_/*N* (coefficient − 1.08, *p* = 0.0072) indicates that this effect becomes even stronger with unfettered migration.

Looking across these statistical interactions, it appears that removing the constraint of vector‐borne transmission makes the direct effects of selection in each trophic level less consequential (it reduces the importance of *ω*_*h*_, *ω*_*v*_), and makes parameters relating to population structure (*N*_*h*_/*N*, *m*, *v*_*max*_) and the interactions between population structure and local selection (*δ*:*ω*_*v*_:*N*_*h*_/*N*) more consequential. Forcing pathogen dispersal between different habitat types makes gene flow between habitat types more even then it would be otherwise, and this makes divergent selection between habitat types a more powerful driver of evolutionary dynamics.

**FIGURE 6:**
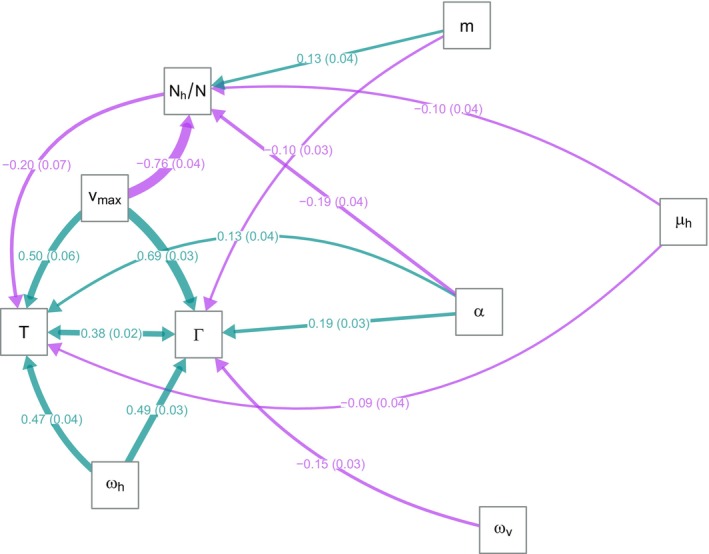
Structured equation analysis of model of the evolution of virulence in a vector‐borne pathogen. Edges show positive (teal) and negative (lavender) causal relationships among model parameters (*ω*_*h*_, *ω*_*v*_, *m*, *v*_*max*_, *α*) and variables (*N*_*h*_/*N*, *T*, *Γ*). The width of each edge is in proportion to the magnitude of its effect. Effect coefficients are printed on each edge, followed by its standard error in parentheses.

We now put everything together in a structural equation model. Figure 6 shows the significant causal relationships for simulations under strong antagonistic pleiotropy (*κ* = −0.8). Note that the parameters *v*_*max*_, *α*, *μ*_*h*_, and *m* affect the evolution of virulence both directly and indirectly via effects on the preponderance of the host environment, *N*_*h*_ */N*. Increasing *v*_*max*_ or *α* directly affects *T* and *Γ* by elevating the rate of virulence‐induced host death, which pushes for sub‐optimal (and virulence‐limiting) within‐host performance phenotypes. It also affects *T* and *Γ* indirectly, by reducing the expected preponderance of the host environment, which reduces the disparity in the efficiency of selection between hosts and vectors. Likewise, increasing the background rate of host mortality, *μ*_*h*_, decreases the meta‐population cost of high virulence and weakens group‐level selection against an optimal within‐host performance phenotype. It also reduces the preponderance of the host environment, and attenuates differences in the efficiency of selection across hosts and vectors. Conversely, increasing the per‐capita transmission rate, *m*, increases the rate of host patch colonization, which increases the preponderance of the host environment and exacerbates differences across hosts and vectors in the efficiency of selection, while also reducing the fitness cost of high virulence. A similar mix of direct and indirect effects has been observed in certain epidemiological compartment models of virulence evolution in a heterogeneous host environment (Williams 2012), although in that case, the indirect effects relate to how changes in model parameters affect the relative abundance of different types of hosts, and thus changes the weighting of host types used to calculate a pathogen's basic reproductive number (Gandon 2004; Williams 2012).

What does the structured equation model reveal about the possibility of non‐adaptive correlational virulence evolution in a vector‐borne pathosystem? Although increasing *ω*_*v*_ does not significantly affect *T*, it does significantly reduce *Γ* (coefficient = −0.16 SD; *p* < 1e‐4); stronger selection in vectors causes the pathogen population's evolution path to deviate more toward the vector optimum, but does not affect the time it takes to evolve a within‐host performance phenotype that is a close match to the within‐host optimum. In contrast, the preponderance of the host environment is an important determinant of the rate of pathogen adaptation to the host, but not of the deviation from the ideal evolutionary path; Larger values for *N*_*h*_ */N* significantly reduce *T* (coefficient = −0.2 SD; *p* = 2e‐3) but not *Γ*. The preponderance of the host habitat, *N*_*h*_ */N*, has a powerful effect on the probability of the evolution of high virulence, but selection in vectors tends to cause more significant deviations from the ideal evolutionary path.

Our inferences are contingent of the many simplifying assumptions of our model, for example, that mutations affecting performance phenotypes are uniformly spread across a non‐recombining genome, and there is no variation across hosts and vectors in time and space. Here we stress three of our most liberal assumptions. The first is the assumption of instantaneous replacement of host and vector patches. Relaxation of this assumption, and allowing for more realistic recruitment, would reduce the preponderance of host patches, and therefore reduce the demographic asymmetries that interfere with selection in vectors. This bias should be counterbalanced to some extent by a second simplifying assumption of our model, namely, that pathogens cause no harm to their vectors, despite some empirical evidence to the contrary (Lambrechts and Scott 2009; Chapuis, Arnal, and Ferdy 2012). A third major assumption in our model is that within‐host and within‐vector performance phenotypes evolve exclusively via pleiotropic alleles. This was integral to our premise; we wondered if antagonistic pleiotropy could suffice to drive correlational evolution of virulence in hosts. Nevertheless, further study of genetic architectural variation could be useful, as many general questions have yet to be answered about how adaptation to heterogeneous environments depends on the genetic architectures of the traits under divergent selection (Kimbrell and Holt 2007; Kawecki 2008; Bridle, Kawata, and Butlin 2019).

## 4 Conclusions

Our goal was to better understand how the population structure and life history of vector‐borne plant pathogens might amend the general rule that selection is more efficient in more common and productive habitats (Via and Lande 1985; Whitlock 1996; Hardy and Forister 2023). More specifically, we wanted to evaluate the plausibility of the hypotheses that the short‐term evolution of virulence in hosts could be a correlational response driven by selection for improved performance in vectors. With statistical analyses of the dynamics of individual‐based simulation models, we were unable to reject this hypothesis. Even when pathogens are much more often found within hosts than vectors, if selection in vectors is sufficiently strong, it can drive correlational evolution in hosts, as measured by our statistic *Γ*. On the other hand, we also recovered evidence of important governing roles for the asymmetries in habitat productivity, and virulence per se that are consistent with previous theory (Via and Lande 1985; Regoes, Nowak, and Bonhoeffer 2000; Day and Proulx 2004; Gandon 2004; Alizon et al. 2009; Evensen, White, and Boots 2024). Strong selection in vectors is one among many factors that can drive the evolution of virulence in hosts.

To close, let us reconsider the evolution of virulence in *Xylella fastidiosa* . In California vineyards, the emergence of new highly virulent genotypes closely followed the establishment of a new and markedly inefficient vector species (Hopkins 1989). Could this have been because of negative genetic correlations between traits affecting performance within vectors and hosts (Gilbertson et al. 2015)? Our analysis suggests that it might have been. Strong selection for improved performance in the vector does seem capable, in at least certain situations, of causing correlational evolution of pleiotropic host performance traits. (In theory this could also arise via tight linkage rather than pleiotropy per se (Via and Lande 1985)). If such correlational evolution in the host causes a non‐adaptive increase in virulence, the demographic consequences could further bend the evolutionary path toward the vector optimum (Gandon 2004; Osnas and Dobson 2012; Williams 2012), and further interfere with overall life‐history optimization.

Of course there are other tenable hypotheses for the evolution of increased virulence in *X. fastidiosa* in Californian vineyards. In particular, in addition to being an especially poor vector, *H. vitripenis* is also exceptionally polyphagous. The story of virulence evolution in Californian populations of *X. fastidiosa* likely also entails changes in their population structure, perhaps increasing the alpha diversity of pathogen communities and the potential for phenotypic evolution via recombination (Gilbertson et al. 2015). Nevertheless, we cannot yet reject the hypothesis that much of the evolution of within‐host virulence can be traced back to selection in vectors.

## Author Contributions

**Nate B. Hardy:** conceptualization (lead), formal analysis (equal), funding acquisition (lead), investigation (equal), methodology (equal), project administration (lead), software (equal), supervision (lead), visualization (equal), writing – original draft (supporting), writing – review and editing (lead). **Elise Woodruff:** conceptualization (supporting), formal analysis (equal), investigation (equal), methodology (equal), resources (equal), software (equal), writing – original draft (lead), writing – review and editing (supporting).

## Conflicts of Interest

The authors declare no conflicts of interest.

## Supporting information


Data S1.

## Data Availability

This work is based on analyses of the dynamics of evolutionary simulation models. The code for these models is provided as a Supporting Information document.

## References

[ece370741-bib-0001] Alizon, S. , A. Hurford , N. Mideo , and M. Van Baalen . 2009. “Virulence Evolution and the Trade‐Off Hypothesis: History, Current State of Affairs and the Future.” Journal of Evolutionary Biology 22, no. 2: 245–259. 10.1111/j.1420-9101.2008.01658.x. 19196383

[ece370741-bib-0002] Anderson, R. M. , and R. M. May . 1982. “Coevolution of Hosts and Parasites.” Parasitology 85, no. 2: 411–426. 10.1017/S0031182000055360. 6755367

[ece370741-bib-0003] Auld, S. K. J. R. , C. L. Searle , and M. A. Duffy . 2017. “Parasite Transmission in a Natural Multihost–Multiparasite Community.” Philosophical Transactions of the Royal Society, B: Biological Sciences 372, no. 1719: 20160097. 10.1098/rstb.2016.0097. PMC5352823 28289264

[ece370741-bib-0004] Bates, D. 2003. “lme4: Linear Mixed‐Effects Models using ‘Eigen’ and S4.” 10.32614/CRAN.package.lme4.

[ece370741-bib-0005] Best, A. , A. White , and M. Boots . 2009. “The Implications of Coevolutionary Dynamics to Host–Parasite Interactions.” American Naturalist 173, no. 6: 779–791. 10.1086/598494. 19374557

[ece370741-bib-0006] Bridle, J. R. , M. Kawata , and R. K. Butlin . 2019. “Local Adaptation Stops Where Ecological Gradients Steepen or Are Interrupted.” Evolutionary Applications 12, no. 7: 1449–1462. 10.1111/eva.12789. 31417626 PMC6691213

[ece370741-bib-0007] Bull, J. J. , and A. S. Lauring . 2014. “Theory and Empiricism in Virulence Evolution.” PLoS Pathogens 10, no. 10: e1004387. 10.1371/journal.ppat.1004387. 25340792 PMC4207818

[ece370741-bib-0008] Bull, J. J. , I. J. Molineux , and W. R. Rice . 1991. “Selection of Benevolence in A Host–Parasite System.” Evolution 45, no. 4: 875–882. 10.1111/j.1558-5646.1991.tb04356.x. 28564051

[ece370741-bib-0009] Bürger, R. , and M. Lynch . 1995. “Evolution and Extinction in a Changing Environment: A Quantitative Genetic Analysis.” Evolution 49, no. 1: 151–163. 10.1111/j.1558-5646.1995.tb05967.x. 28593664

[ece370741-bib-0010] Chapuis, É. , A. Arnal , and J.‐B. Ferdy . 2012. “Trade‐offs Shape the Evolution of the Vector‐Borne Insect Pathogen *Xenorhabdus nematophila* .” Proceedings of the Royal Society B: Biological Sciences 279, no. 1738: 2672–2680. 10.1098/rspb.2012.0228. PMC3350712 22398163

[ece370741-bib-0011] Cressler, C. E. , D. V. McLeod , C. Rozins , J. Van Den Hoogen , and T. Day . 2016. “The Adaptive Evolution of Virulence: A Review of Theoretical Predictions and Empirical Tests.” Parasitology 143, no. 7: 915–930. 10.1017/S003118201500092X. 26302775 PMC4873896

[ece370741-bib-0012] Crossan, J. , S. Paterson , and A. Fenton . 2007. “Host Availability and the Evolution of Parasite Life‐History Strategies.” Evolution 61, no. 3: 675–684. 10.1111/j.1558-5646.2007.00057.x. 17348930

[ece370741-bib-0013] Day, T. 2001. “Parasite Transmission Modes and the Evolution of Virulence.” Evolution 55, no. 12: 2389–2400. 10.1111/j.0014-3820.2001.tb00754.x. 11831655

[ece370741-bib-0014] Day, T. 2003. “Virulence Evolution and the Timing of Disease Life‐History Events.” Trends in Ecology & Evolution 18, no. 3: 113–118. 10.1016/S0169-5347(02)00049-6.

[ece370741-bib-0015] Day, T. , and S. R. Proulx . 2004. “A General Theory for the Evolutionary Dynamics of Virulence.” American Naturalist 163, no. 4: E40–E63. 10.1086/382548. 15122509

[ece370741-bib-0016] Dieckmann, U. , ed. 2005. “Digitally Printed First Paperback Edition.” In Adaptive Dynamics of Infectious Diseases: In Pursuit of Virulence Management, 2. Cambridge New York Melbourne: Cambridge University Press (Cambridge studies in adaptive dynamics).

[ece370741-bib-0017] Dobson, A. P. 1990. “Models for Multi‐Species Parasite–Host Communities.” In Parasite Communities: Patterns and Processes, edited by G. W. Esch , A. O. Bush , and J. M. Aho , 261–288. Dordrecht: Springer Netherlands. 10.1007/978-94-009-0837-6_10.

[ece370741-bib-0018] Draghi, J. A. 2021. “Asymmetric Evolvability Leads to Specialization Without Trade‐offs.” American Naturalist 197, no. 6: 644–657. 10.1086/713913. 33989145

[ece370741-bib-0019] Ebert, D. , and K. L. Mangin . 1997. “The Influence of Host Demography on the Evolution of Virulence of a Microsporidian Gut Parasite.” Evolution 51, no. 6: 1828–1837. 10.1111/j.1558-5646.1997.tb05106.x. 28565099

[ece370741-bib-0020] Evensen, C. , A. White , and M. Boots . 2024. “Multispecies Interactions and the Community Context of the Evolution of Virulence.” Proceedings of the Royal Society B: Biological Sciences 291, no. 2031: 20240991. 10.1098/rspb.2024.0991. PMC11421928 39317313

[ece370741-bib-0021] Ewald, P. W. 1983. “Host–Parasite Relations, Vectors, and the Evolution of Disease Severity.” Annual Review of Ecology and Systematics 14, no. 1: 465–485. 10.1146/annurev.es.14.110183.002341.

[ece370741-bib-0022] Frank, S. A. 1996. “Models of Parasite Virulence.” Quarterly Review of Biology 71, no. 1: 37–78. 10.1086/419267. 8919665

[ece370741-bib-0023] Gandon, S. 2004. “Evolution of Multihost Parasites.” Evolution 58, no. 3: 455–469. 10.1111/j.0014-3820.2004.tb01669.x. 15119430

[ece370741-bib-0024] Geritz, S. A. H. , É. Kisdi , G. Meszéna , and J. A. J. Metz . 1998. “Evolutionarily Singular Strategies and the Adaptive Growth and Branching of the Evolutionary Tree.” Evolutionary Ecology 12, no. 1: 35–57. 10.1023/A:1006554906681.

[ece370741-bib-0025] Gilbertson, R. L. , O. Batuman , C. G. Webster , and S. Adkins . 2015. “Role of the Insect Supervectors Bemisia Tabaci and *Frankliniella occidentalis* in the Emergence and Global Spread of Plant Viruses.” Annual Review of Virology 2: 67–93. 10.1146/annurev-virology-031413-085410. 26958907

[ece370741-bib-0026] Haller, B. C. , and P. W. Messer . 2023. “SLiM 4: Multispecies Eco‐Evolutionary Modeling.” American Naturalist 201, no. 5: E127–E139. 10.1086/723601. PMC10793872 37130229

[ece370741-bib-0027] Hardy, N. B. , and M. L. Forister . 2023. “Niche Specificity, Polygeny, and Pleiotropy in Herbivorous Insects.” American Naturalist 201, no. 3: 376–388. 10.1086/722568. 36848511

[ece370741-bib-0028] Hopkins, D. L. 1989. “ *Xylella fastidiosa* : Xylem‐Limited Bacterial Pathogen of Plants.” Annual Review of Phytopathology 27: 271–290. 10.1146/annurev.phyto.34.1.131 15012538

[ece370741-bib-0046] Hopkins, D. L. and A. H. Purcell . 2002. “ *Xylella fastidiosa* : Cause Of Pierce’s Disease of Grapevine and Other Emergent Diseases.” Plant Disease 86, no. 10: 1056–1066. 30818496 10.1094/PDIS.2002.86.10.1056

[ece370741-bib-0029] Kakehashi, M. , and F. Yoshinaga . 1992. “Evolution of Airborne Infectious Diseases According to Changes in Characteristics of the Host Population.” Ecological Research 7, no. 3: 235–243. 10.1007/BF02347092.

[ece370741-bib-0030] Kawecki, T. J. 2008. “Adaptation to Marginal Habitats.” Annual Review of Ecology, Evolution, and Systematics 39, no. 1: 321–342. 10.1146/annurev.ecolsys.38.091206.095622.

[ece370741-bib-0047] Killiny, N. and R. P. Almeida . 2014. “Factors Affecting The Initial Adhesion and Retention of the Plant Pathogen *Xylella fastidiosa* In the Foregut of an Insect Vector.” Applied and environmental microbiology 80, no. 1: 420–426. 24185853 10.1128/AEM.03156-13 PMC3910991

[ece370741-bib-0031] Kimbrell, T. , and R. D. Holt . 2007. “Canalization Breakdown and Evolution in a Source‐Sink System.” American Naturalist 169, no. 3: 370–382. 10.1086/511314. 17294371

[ece370741-bib-0032] Lambrechts, L. , and T. W. Scott . 2009. “Mode of Transmission and the Evolution of Arbovirus Virulence in Mosquito Vectors.” Proceedings of the Royal Society B: Biological Sciences 276, no. 1660: 1369–1378. 10.1098/rspb.2008.1709. PMC2660968 19141420

[ece370741-bib-0033] Lenski, R. E. , and R. M. May . 1994. “The Evolution of Virulence in Parasites and Pathogens: Reconciliation Between Two Competing Hypotheses.” Journal of Theoretical Biology 169, no. 3: 253–265. 10.1006/jtbi.1994.1146. 7967617

[ece370741-bib-0034] Longdon, B. , J. D. Hadfield , J. P. Day , et al. 2015. “The Causes and Consequences of Changes in Virulence Following Pathogen Host Shifts.” PLoS Pathogens 11, no. 3: e1004728. 10.1371/journal.ppat.1004728. 25774803 PMC4361674

[ece370741-bib-0035] Ni, Y. , and M. C. Kemp . 1992. “Strain‐Specific Selection of Genome Segments in Avian Reovirus Coinfections.” Journal of General Virology 73, no. 12: 3107–3113. 10.1099/0022-1317-73-12-3107. 1469350

[ece370741-bib-0036] Osnas, E. E. , and A. P. Dobson . 2012. “Evolution of Virulence in Heterogeneous Host Communities Under Multiple Trade‐offs.” Evolution 66, no. 2: 391–401. 10.1111/j.1558-5646.2011.01461.x. 22276536

[ece370741-bib-0037] Redak, R. A. , A. H. Purcell , J. R. S. Lopes , M. J. Blua , R. F. Mizell III , and P. C. Andersen . 2004. “The Biology of Xylem Fluid‐Feeding Insect Vectors of *Xylella fastidiosa* and Their Relation to Disease Epidemiology.” Annual Review of Entomology 49: 243–270. 10.1146/annurev.ento.49.061802.123403. 14651464

[ece370741-bib-0038] Regoes, R. R. , M. A. Nowak , and S. Bonhoeffer . 2000. “Evolution of Virulence in a Heterogeneous Host Population.” Evolution 54, no. 1: 64–71. 10.1111/j.0014-3820.2000.tb00008.x. 10937184

[ece370741-bib-0039] Rosseel, Y. 2012. “lavaan: An R Package for Structural Equation Modeling.” Journal of statistical software 48: 1–36.

[ece370741-bib-0040] Scheiner, S. M. 2013. “The Genetics of Phenotypic Plasticity. XII. Temporal and Spatial Heterogeneity.” Ecology and Evolution 3, no. 13: 4596–4609. 10.1002/ece3.792. 24340198 PMC3856757

[ece370741-bib-0041] Scheiner, S. M. , and R. D. Holt . 2012. “The Genetics of Phenotypic Plasticity. X. Variation Versus Uncertainty.” Ecology and Evolution 2, no. 4: 751–767. 10.1002/ece3.217. 22837824 PMC3399198

[ece370741-bib-0042] Via, S. , and R. Lande . 1985. “Genotype‐Environment Interaction and the Evolution of Phenotypic Plasticity.” Evolution 39, no. 3: 505–522. 10.1111/j.1558-5646.1985.tb00391.x. 28561964

[ece370741-bib-0043] Whitlock, M. C. 1996. “The Red Queen Beats the Jack‐Of‐All‐Trades: The Limitations on the Evolution of Phenotypic Plasticity and Niche Breadth.” American Naturalist 148: S65–S77. 10.1086/285902.

[ece370741-bib-0044] Williams, P. D. 2012. “New Insights Into Virulence Evolution in Multigroup Hosts.” American Naturalist 179, no. 2: 228–239. 10.1086/663690. 22218312

[ece370741-bib-0045] Williams, P. D. , and T. Day . 2008. “Epidemiological and Evolutionary Consequences of Targeted Vaccination.” Molecular Ecology 17, no. 1: 485–499. 10.1111/j.1365-294X.2007.03418.x. 18173510

